# Effects of enzymolysis and fermentation of Chinese herbal medicines on serum component, egg production, and hormone receptor expression in laying hens

**DOI:** 10.5713/ab.23.0146

**Published:** 2023-10-27

**Authors:** Mei Hong Jiang, Tao Zhang, Qing Ming Wang, Jin Shan Ge, Lu Lu Sun, Meng Qi Li, Qi Yuan Miao, Yuan Zhao Zhu

**Affiliations:** 1 Key Laboratory for Animal Nutritional Regulation and Health of the Anhui Province, College of Animal Science, Anhui Science and Technology University, Bengbu 233100, China; 2 Shandong Jinghua Agriculture and Animal Husbandry Development Co., Ltd., Zhucheng 262200, China; 3 Shandong Zhongcheng Feed Technology Co., Ltd., Feicheng 271600, China

**Keywords:** Chinese Herbal Medicines, Enzymolysis and Fermentation, Laying Hens, Lipid Metabolism, Productive Performance, Reproductive Hormones

## Abstract

**Objective:**

In the present study, we aimed to investigate the effects of enzymolysis fermentation of Chinese herbal medicines (CHMs) on egg production performance, egg quality, lipid metabolism, serum reproductive hormone levels, and the mRNA expression of the ovarian hormone receptor of laying hens in the late-laying stage.

**Methods:**

A total of 360 Hy-Line Brown laying hens (age, 390 days) were randomly categorized into four groups. Hens in the control (C) group were fed a basic diet devoid of CHMs, the crushed CHM (CT), fermented CHM (FC), and enzymatically fermented CHM (EFT) groups received diets containing 2% crushed CHM, 2% fermented CHM, and 2% enzymatically fermented CHM, respectively.

**Results:**

Compared with crushed CHM, the acid detergent fiber, total flavonoids, and total saponins contents of fermented CHM showed improvement (p<0.05); furthermore, the neutral and acid detergent fiber, total flavonoids, and total saponins contents of enzymatically fermented CHM improved (p<0.05). At 5 to 8 weeks, hens in the FC and EFT groups showed increased laying rates, haugh unit, albumin height, yolk color, shell thickness, and shell strength compared with those in the C group (p<0.05). Compared with the FC group, the laying rate, albumin height, and Shell thickness in the EFT group was increased (p<0.05). Compared with the C, CT, and FC groups, the EFT group showed reduced serum total cholesterol and increased serum luteinizing hormone levels and mRNA expressions of follicle stimulating hormone receptor and luteinizing hormone receptor (p<0.05).

**Conclusion:**

These results indicated that the ETF group improved the laying rate and egg quality and regulated the lipid metabolism in aged hens. The mechanism underlying this effect was likely related to cell wall degradation of CHM and increased serum levels of luteinizing hormone and mRNA expression of the ovarian hormone receptor.

## INTRODUCTION

The late egg-laying period is characterized by the gradual deterioration of lipid metabolic functions, which contributed to decreased egg production performance and potential health problems in laying hens [1]. Furthermore, the luteinizing hormone (LH) and ovarian hormone receptor mRNA expression directly affected egg-laying rates in hens [2]. Therefore, safe feed additives to maintain egg production performance during the late-laying period are necessary.

Chinese herbal medicines (CHMs) have bacteriostatic, antiviral, and immune-enhancing effects and their merits are that they are safe, effective, and abundant [3]. The use of CHM is a whole concept, single CHM in practical application cannot reach the effect. Multiple interactions of CHMs are needed to enhance their effectiveness. *Astragalus* and *Angelica sinensis* predominantly improve the weakened cardiovascular function caused by aging and enhance immunity. *Alpinia oxyphylla* and *Wolfiporia extensa Ginns* can tonify kidney and spleen, and strengthen the regulation of the whole function. After adequately improving the body functions, *Leonurus japonicus* acts on reproductive organs and regulates egg production performance. *Chinese hawthorn* and dandelion can increase appetite, harmonize the nature of the drug, and reduce the toxicity of the formula. CHMs predominantly function via the effects of intracellular active ingredients to balance the internal environment, unblock the Zhangfu organ meridians, inhibit pathogens, and control diseases [4]. However, total flavonoids, crude polysaccharides, and other active ingredients may not be completely released because they are protected by the hard cell wall [5], which reduces the efficacy. Therefore, the enzymatic fermentation of CHMs has garnered considerable attention. Enzymolysis fermentation are performed using CHMs as a substrate, with the addition of compound enzymes and probiotics for synergistic fermentation. In the enzymatically fermented CHM system, enzymes can degrade cellulose, hemicellulose, and pectin in the cell walls of CHMs [6], thereby releasing intracellular active ingredients. Nonetheless, the degradation ability of enzymes is related to the enzyme source and its combinations as well as whether the fermentation environment is conducive to enzymatic action. Probiotics in the fermentation system can reportedly proliferate rapidly, thereby inhibiting pathogenic bacteria and improving health [7]. Furthermore, these probiotics can produce small peptides, amino acids, oligosaccharides, immune factors, and other microbial secondary metabolites during the fermentation process [8,9]. Therefore, enzymatic action and fermentation reduce the bitterness of CHMs, improve their palatability, reduce their adverse effects, enhance their efficacy, and help in improving nutrient absorption from the feed [10].

Currently, studies on the enzymolysis fermentation of CHMs to improve the late-laying performance of hens and understand the underlying mechanism are lacking. Tian et al [11] reported that adding fermented Chinese herbs to the feed significantly increased production performance and egg quality of laying hens and reduced production costs. Moreover, most studies are based on the anaerobic fermentation of single or compound CHMs with no addition of synergistic exogenous enzymes. The mechanism underlying the effects of fermented CHMs remains unclear. However, the different CHM compounds show different efficacies, and combined fermentation by multiple bacterial species is more beneficial to the health of the organism than fermentation by a single bacterial species [12]. Therefore, in this experiment, according to the principles of incompatibility and compensation multiplication, we rationally formulated compound CHMs to improve reproductive function. We screened compound probiotics and high-cleavage compound enzymes, performed anaerobic fermentation, and established an efficient and synergistic enzymatic fermentation system. We studied the effects of cell wall degradation and release of CHM active components during enzymolysis fermentation on egg production performance, egg quality, lipid metabolism, serum reproductive hormone levels, and the mRNA expression of the follicle stimulating hormone receptor (FSHR), luteinizing hormone receptor (LHR), and estrogen receptor 2 (ESR2) in laying hens during the late-laying period.

## MATERIALS AND METHODS

### Experimental materials

The composition and content of crushed CHM, fermented CHM, and enzymatically fermented CHM are shown in Table 1. Compound CHMs, corn, wheat bran, soybean meal, and brown sugar were crushed and sieved through a 40-mesh screen. Compound CHMs comprised *Astragalus*, *Chinese hawthorn*, *Wolfiporia extensa Ginns*, *Angelica sinensis*, *Leonurus japonicus*, *Alpinia oxyphylla*, and dandelion, which were mixed in the ratio of 4:4:2:4:3:2:6. The raw CHM materials were purchased from Bozhou Hongzhe Pharmaceutical Co., Ltd. The enzyme activities of cellulase, xylanase, pectinase, and phytase were 1×10^4^ U/g, 3×10^4^ U/g, 3×10^4^ U/g, and 4×10^4^ U/g, respectively, and they were purchased from Xin Dayang Neiqiu Biology Science and Technology Co., Ltd. *Lactobacillus plantarum*, *Saccharomyces cerevisiae*, and *Aspergillus niger* live bacterial counts were 1×10^10^ CFU/g, 2×10^10^ CFU/g, and 2×10^11^ CFU/g, respectively. They were purchased from Jiangsu Youshi Biotechnology Development Co., Ltd.

### Preparation of crushed Chinese herbal medicine, fermented Chinese herbal medicine, and enzymatically fermented Chinese herbal medicine

Crushed CHM was mixed according to the material ratio given in Table 1. Fermented CHM and enzymatically fermented CHM were prepared according to the material composition specified in Table 1. First, *Lactobacillus plantarum*, *Saccharomyces cerevisiae*, and *Aspergillus niger* were added to warm water (40°C) and left for 30 min for activation. Subsequently, the activated bacterial suspension was inoculated into the prepared substrates and mixed uniformly. The mixture was subjected to anaerobic fermentation at 37°C for 7 d in a fermentation bag with a one-way breather valve to prepare the fermented CHM and enzymatically fermented CHM. Six replicates were performed for crushed CHM, fermented CHM, and enzymatically fermented CHM.

### Animals and experimental design

A total of 360 healthy 390-day-old Hy-Line Brown laying hens with initial laying rate of 84%±1% were randomly allocated to four groups of six replicates each, with 15 hens in each replicate, and reared in cages (5 hens per cage). The control (C) group was fed a basal diet. The crushed CHM (CT), fermented CHM (FC), and enzymatically fermented CHM (EFT) groups were fed an experimental group diet containing 2% crushed CHM, 2% fermented CHM, and 2% enzymatically fermented CHM, respectively. The diet formulations and nutrient levels are shown in Table 2. Before feeding, the prepared crushed CHM, fermented CHM, or enzymatically fermented CHM were premixed with soybean meal and then mixed with other raw materials in Table 2. The laying hens were given *ad libitum* access to feed and water during the experiment and fed twice a day at 07:00 and 15:00 for 8 weeks. The experimental protocol of this study was approved by the Animal Care Committee of the Anhui Science and Technology University (2020-05).

### Cell wall fibers and intracellular active ingredients of Chinese herbal medicine

The levels of neutral detergent fiber (NDF), acid detergent fiber (ADF), total flavonoids, crude polysaccharides, and total saponins were measured crushed CHM, fermented CHM, and enzymatically fermented CHM. NDF and ADF were measured sequentially [13] using an Fiber Analyzer (ANKOM 220; ANKOM, Macedon, NY, USA). Total flavonoids, crude polysaccharides, and total saponins were determined using the NaNO_2_–Al(NO_3_)_3_–NaOH colorimetric method, phenol–sulfuric acid method, and vanillin–perchloric acid colorimetric method, respectively [14].

### Egg production performance, egg quality, and sampling

The number of eggs and egg weight for each replicate was recorded daily, and the feed intake of each replicate was recorded weekly. The feed conversion ratio (FCR) was calculated as feed intake divided by the total egg weight. In each group, 30 eggs were randomly tested per week for egg quality using an egg analyzer (EA-01; Tenovo International Co., Ltd, Beijing, China) and an egg force reader (RH-DQ200; Guangzhou Runhu Instrument Co., Ltd, Guangzhou, China). At the end of the experiment, 12 hens were randomly selected from each group, and 10 mL of blood was collected from the wing vein after 12 h of fasting. The blood was centrifuged at 4°C and 3,000 rpm for 15 min. Serum was collected and stored at −20°C. Following blood collection and euthanasia, the ovaries were removed and stored at −80°C after liquid nitrogen snap-freezing.

### Serum lipid metabolism

Total cholesterol (TC), triglyceride (TG), and alkaline phosphatase (ALP) levels in the serum were measured using an automatic biochemistry analyzer (ZY-350; Kehua Biotechnology Co., Ltd, Shanghai, China). The reagent kits were purchased from Shanghai Kehua Bio-Engineering Co. Ltd. (Shanghai, China).

### Serum reproductive hormones

Serum levels of estrogen (E2), follicle stimulating hormone (FSH), and LH were measured using an automatic enzyme-linked immunosorbent assay system (Multiskan Go, Waltham, MA, USA). The reagent kits were procured from Beijing Daka Mei Technology Co., Ltd. (Beijing, China).

### Ovarian hormone receptor mRNA expression

Ovarian hormone mRNA was extracted using the TRIzol method, and the RNA concentration was determined using a nucleic acid protein analyzer (Nanodrop One; Thermo, Waltham, MA, USA). RNA quality was assessed with 3% agarose gel electrophoresis and reverse transcribed to cDNA using a kit from Tiangen Biotechnology Co., Ltd. (Beijing, China). Primers were designed using the software Primer Premier 5 for the gene sequences of the FSHR, LHR, ESR2, and β-actin. The primers were synthesized by Sangon Biotech (Shanghai) Co., Ltd. (Shanghai, China). The primer information is provided in Table 3. FSHR, LHR, and ESR2 were quantitatively analyzed with SYBR Green quantitative real-time polymerase chain reaction (ABI-7300; ABI, Carlsbad, CA, USA), and the kits were obtained from Tiangen Biotechnology Co., Ltd. (Beijing, China). Relative gene expression was calculated using the 2^−ΔΔCt^ method, with β-actin serving as the reference gene.

### Statistical analysis

SPSS software (version 21.0; IBM Inc., San Francisco, CA, USA) was used for one-way analysis of variance followed by Duncan’s multiple comparison tests of the experimental data. The results were expressed as means. A difference of p<0.05 was considered to be significant.

## RESULTS

### Changes in fiber and active ingredient content Chinese herbal medicine

Changes in fiber and active ingredient concentrations in different treatment methods of CHM are shown in Table 4. NDF levels were lower (p = 0.003) and the total flavonoids and total saponin were higher (p<0.001) in the enzymatically fermented CHM than in the crushed CHM and fermented CHM. The enzymatically fermented CHM and fermented CHM had lower ADF levels than crushed CHM (p = 0.022). NDF levels were lower (p = 0.003) and the total saponin levels were higher (p<0.001) in the enzymatically fermented CHM than in the fermented CHM.

### Egg production performance

The effects of different treatment methods of CHM on egg production performance of laying hens in the late-laying period are shown in Table 5. During the 1 to 8 weeks of the experiment, the laying rate was higher and the FCR was lower in the FC and EFT groups than in the C and CT groups (p< 0.05). During the 5 to 8 weeks of the experiment, the CT, FC, and EFT groups had higher feed intake than C group (p<0.001). The EFT group had higher laying rate than the FC group (p<0.001). The EFT group had a lower FCR than CT group (p<0.001).

### Egg quality

The effects of different treatment methods of CHM on egg quality of laying hens in the late-laying period are shown in Table 6. During the 1 to 4 weeks of the experiment, the ETF group had a thicker of eggshell than the C group (p<0.034). During the 5 to 8 weeks of the experiment, the haugh unit (p = 0.009) and albumin height (p<0.001) were higher in the FC and EFT groups than in the C and CT groups. The yolk color was more intense in the CT, FC, and EFT groups than in the C group (p = 0.006). The eggshell thickness (p<0.001) and shell strength (p = 0.012) were greater in the FC and EFT groups than in the C group. The ETF group had greater albumin height and eggshell thickness than the C group (p<0.001).

### Serum lipid metabolism and serum reproductive hormones

As shown in Table 7, the serum TC levels were lower in the FC and EFT groups than in the C and CT groups (p<0.001). The ETF group had lower serum TC levels than the FC group (p<0.001). Furthermore, serum TG and ALP levels showed no significant intergroup differences. As shown in Table 8, serum E2 and FSH levels were not significantly different among all groups. The ETF group had higher serum LH levels than the C, CT, and FC groups (p<0.001).

### Ovarian hormone receptor mRNA expression

As presented in Table 9, the mRNA expression levels of FSHR, LHR, and ESR2 were higher in the FC and EFT groups than in the C and CT groups (p<0.001). The CT group had higher mRNA expression of FSHR and LHR than the C group (p< 0.001). The ETF group had higher mRNA expression of FSHR and LHR than the FC group (p<0.001).

## DISCUSSION

Different types of CHMs and their combinations have different effects. The seven CHMs selected in this experiment, namely, *Astragalus*, *Chinese hawthorn*, *Wolfiporia extensa Ginns*, *Angelica sinensis*, *Leonurus japonicus*, *Alpinia oxyphylla*, and dandelion, can strengthen the spleen, dispel dampness, activate blood, exert an antiinflammatory effect, and enhance immunity. Most of the active ingredients in the CHM are protected by hard cell walls that cannot be easily degraded, affecting the release and efficacy of the ingredients. In this experiment, the contents of NDF and ADF in enzymatically fermented CHM were significantly decreased by 10.39% and 12.70%, respectively, compared with crushed CHM. The contents of NDF in enzymatically fermented CHM were significantly decreased by 17.54%, compared with fermented CHM. The contents of ADF in fermented CHM were significantly decreased by 8.99%, compared with crushed CHM. The results showed that the fiber could be degraded by enzymolysis fermentation or fermentation. However, after enzymolysis fermentation, CHM released more cell contents and cell wall lysed more thoroughly. These observations were verified by another result of this experiment, which found that the total flavonoid and saponin contents of the enzymatically fermented CHM were significantly increased by 50.00% and 42.62%, respectively, compared with the crushed CHM. The total flavonoid and saponin contents of the fermented CHM were significantly increased by 50.00% and 14.75%, respectively, compared with the crushed CHM. This may be because the probiotics added in the fermentation CHM can metabolize enzymes, but the activity of enzymes is low and the types are not comprehensive enough, which leads to insufficient fermentation. Cellulase, xylanase, pectinase, and phytase added to the enzymatically fermented CHM system can dissolve the cell wall components more thoroughly [15]. Liu et al [16] used *Candida utilis*, *Lactobacillus casei*, and *Enterococcus faecalis* to ferment *Saponaria vaccaria* and *Leonurus japonicus*. After fermentation, total flavonoids, total alkaloids, crude polysaccharides, and total saponins increased significantly by 55.14%, 127.28%, 55.42%, and 49.21%, respectively, compared to before fermentation. This result is consistent with the increase in total flavonoids and saponins observed in fermented CHM in this study. Furthermore, the content of crude polysaccharide in this experiment was not significantly improved. It was speculated that the crude polysaccharide contents of different CHMs differed. Another possibility is that the probiotics in the fermentation or enzymolysis fermentation system used a small amount of polysaccharides as substrates, which requires further in-depth studies.

In this study, the crushed CHM did not improve the laying performance and egg quality of the hens. It is hypothesized that the active ingredients of CHM sifted through the 40-mesh sieve after physical pulverization could not be fully released, thus affecting the efficacy of CHM. During the 5 to 8 weeks of this study, the FC and EFT groups showed significant improvements of 5.38% and 8.80%, 10.62% and 12.09%, 2.74% and 4.81%, 2.08% and 2.88%, 3.26% and 4.71%, 3.92% and 7.28%, 5.13% and 7.53% in the laying rate, FCR, haugh unit, albumin height, yolk color, shell thickness, shell strength, respectively, compared with the C group. The CHM enzymatically fermented using compound enzyme and probiotics had a more positive effect on egg production performance and egg quality compared to the crushed CHM and fermented CHM. This can be attributed to the release of more active ingredients, including total flavones and total saponins, by exogenous enzymes acting on the cell wall of CHM during enzymatic fermentation. These active ingredients can interact with probiotics to produce novel active compounds, which can improve the pharmacological properties of CHM [17]. Zhao et al [12] fermented ginkgo leaves with yeast and found no significant effect on the laying rate of hens, whereas fermented ginkgo leaves with *Aspergillus niger* and multiple strains significantly increased the laying rates of hens by 4.66% and 4.07%, respectively, compared with that in the control group. This finding implies that the effect of CHM fermentation on the egg production performance of laying hens is related to the fermentation strain. In this experiment, three strains were selected, of which *Aspergillus niger* and *Saccharomyces cerevisiae* consumed the oxygen in the fermentation substrate, creating an anaerobic environment to facilitate the anaerobic fermentation of *Lactobacillus plantarum* and resulting in a more complete fermentation. Furthermore, the FC and EFT groups had a higher laying rate, which could be because the fermentation or enzymolysis fermentation effect of compound CHMs is better than that of single CHM [18]. Although a single CHM has good efficacy, a compound CHMs acting on different pharmacological targets will produce a synergistic effect that is 50 times greater than that of a single CHM at the same concentration [19].

Prolongation of the production cycle in laying hens leads to gradual aging of the body tissues and decrease in the levels of metabolizable lipids, which affecting egg production performance. In this experiment, serum TC levels in the EFT group were significantly reduced by 30.80%, 30.53%, and 14.15% compared with those in the C, CT, and FC groups, respectively. This could be because dandelion contains total flavonoids such as luteolin 22, which accelerates lipid metabolism [20]. Total flavonoids in *Chinese hawthorn* can also improve liver lipid metabolism [21]. Furthermore, the structural changes of total flavonoids mediated by compound enzyme and probiotic enzymolysis fermentation accelerated the absorption rate and amount of the active ingredients, which may improve the bioactivity and bioavailability of the active ingredients [22,23]. The substrate after enzymolysis can be better utilized by thallus and form a positive promoting effect. However, enzymolysis fermentation of CHM did not significantly reduce serum TG levels, warranting further studies. Serum TC levels were also significantly lower in the FC group than in the C and CT groups. These results indicated that fermentation CHM could also play a role in regulating lipid metabolism; however, the effect was not as good as that of enzymolysis fermentation CHM. This could be because probiotics in the fermentation CHM not only proliferated in large numbers and effectively regulated the balance of intestinal microflora but also produced organic acids and endogenous enzymes capable of inhibiting and killing pathogenic bacteria, promoting the absorption and utilization of nutrients such as lipids [24], facilitating nutrient metabolism, and reducing lipid deposition.

Reproductive hormones in laying hens interact with the hypothalamic–pituitary–gonadal axis and regulate the entire reproductive activity, thus affecting their egg production performance and egg quality [25]. In the CHMs formula of this experiment, *Leonurus japonicas* is rich in leonurine, which can promote the secretion of LH and FSH by the pituitary gland [26]. *Wolfiporia extensa Ginns* is rich in Fuling polysaccharide, which can improve the health and regulate the balance of sex hormones [27]. Ferulic acid in *Angelica sinensis* and total flavonoids in *Astragalus* can restore ovarian function [28]. The above-mentioned CHMs can reduce toxicity and enhance efficacy after enzymolysis fermentation [17]. The serum LH level of laying hens in the EFT group significantly increased by 16.87% compared with that in the C group, but there was no significant increase in the CT and FC groups. This result indicates that enzymolysis fermentation of CHM regulate the reproductive performance of laying hens better than crushed CHM and fermented CHM. This was also confirmed by the results of the laying rate in the above groups in this experiment. Xiao et al [29] showed that when laying hens were given a combination of *Astragalus*, *Salvia miltiorrhiza*, and *Cnidium monnieri*, their serum LH levels increased significantly by 41.63% compared with the control group. The serum LH level in the above study increased more than that in the present study, which may be related to the different formula of CHMs and needs further research. Increased LH levels facilitate the regulation of follicular growth and maturation. Nonetheless, FSH and LH cannot directly penetrate the cell membrane and require the mediation of ovary-specific receptors [30]. Therefore, increased expressions of FSHR and LHR play an important role in reproductive performance. ESR2 acts as a signal transduction factor in the cytoplasm of oocytes to promote their development and maintain ovarian function by regulating steroid hormones [31]. In this experiment, the expressions of FSHR, LHR, and ESR2 mRNA in the ovaries of laying hens in the FC and EFT groups were significantly increased compared with those in the C and CT groups. However, EFT group had better effect. This also demonstrates the triple synergistic effects of cell wall degradation, probiotic fermentation, and the inherent potency of CHM in enzymolysis fermentation. Therefore, the enzymolysis fermentation of CHM have a good delaying effect on the decline in egg production performance and egg quality caused by the prolonged production cycle in late-laying hens. This may be related to the increased expression of serum levels of LH and mRNA expression of FSHR, LHR, and ESR2.

## CONCLUSION

The outcomes of the present study have demonstrated that the CHMs treated with enzymolysis fermentation delay the decline in egg production performance and egg quality and enhance lipid metabolism in the late-laying stage of hens. The mechanisms may be associated with cell wall degradation of CHM by exogenous compound enzymes, increased intracellular release of active components, and an increase in serum LH levels and mRNA expressions of FSHR, LHR, and ESR2.

## Figures and Tables

**Table 1:** Composition and content of crushed Chinese herbal medicine, fermented Chinese herbal medicine, and enzymatically fermented Chinese herbal medicine

Raw material	Crushed CHM	Fermented CHM	Enzymatically fermented CHM
Compound CHM	18.18	18.18	18.18
*Aspergillus niger*	0.00	0.13	0.13
*Saccharomyces cerevisiae*	0.00	0.04	0.04
*Lactobacillus plantarum*	0.00	0.13	0.13
Pectinase	0.00	0.00	0.10
Cellulase	0.00	0.00	0.03
Xylanase	0.00	0.00	0.07
Phytase	0.00	0.00	0.10
Corn	20.36	20.36	20.36
Wheat bran	12.73	12.73	12.73
Soybean meal	8.48	8.48	8.48
Brown sugar	0.85	0.85	0.85
Water	39.40	39.10	38.80
Total	100.00	100.00	100.00

CHM, Chinese herbal medicine.

**Table 2:** Feed formulation and nutrient levels (based on air-dried feed)

Item	C	CT	FC	EFT
Corn	62.50	61.00	61.00	61.00
Soybean meal	24.50	24.00	24.00	24.00
Calcium carbonate	8.00	8.00	8.00	8.00
Premixed feed1)	5.00	5.00	5.00	5.00
Crushed CHM	0.00	2.00	0.00	0.00
Fermented CHM	0.00	0.00	2.00	0.00
Enzymatically fermented CHM	0.00	0.00	0.00	2.00
Total	100.00	100.00	100.00	100.00
Nutrient level				
Crude protein	15.78	15.62	15.61	15.61
Calcium	3.81	3.81	3.81	3.81
Total phosphorus	0.45	0.45	0.45	0.45
Sodium chloride	0.33	0.33	0.33	0.33
Crude ash	13.73	13.72	13.72	13.72
Crude fiber	2.34	2.38	2.38	2.38
Metabolizable energy (MJ/kg)	10.88	10.75	10.75	10.75
Lysine	0.79	0.79	0.79	0.79
Methionine	0.38	0.38	0.38	0.38
Isoleucine	0.59	0.58	0.58	0.58
Threonine	0.65	0.64	0.64	0.64
Tryptophan	0.20	0.20	0.20	0.20
Valine	0.72	0.71	0.71	0.71
Dry matter	88.47	87.82	87.82	87.82

C, control group; CT, crushed Chinese herbal medicine group; FC, fermented Chinese herbal medicine group; EFT, enzymatically fermented Chinese herbal medicine group; CHM, Chinese herbal medicine.

1) The premixed feed consists of the following: Vitamin A 10,000 IU, Vitamin E 25 mg, Vitamin D_3_ 2,500 IU, Vitamin K_3_ 1.0 mg, Vitamin B_1_ 2.5 mg, Vitamin B_2_ 5.5 mg, Vitamin B_6_ 4.0 mg, Vitamin B_12_ 0.008 mg, niacin 31 mg, pantothenic acid 16 mg, biotin 0.3 mg, choline 500 mg, folic acid 1.8 mg, Fe 90 mg, Cu 12.5 mg, Zn 80 mg, Se 0.2 mg, Mn 80 mg, and I 0.45 mg per kg of feed.

**Table 3:** Primer information

Gene	Accession No.	Primer sequence (5′–3′)	Product size (bp)
*FSHR*	NM_205079.2	F: ACCTGCCTGGATGAGCTAAAT R: ATCCAAAACAACAGGCCCGA	96
*LHR*	XM_038176401.1	F:ACTTGAGGACAGAGAACTACAGAG R: TTTCTTCATCTGCTGCAAGCG	93
*ESR2*	NM_001396358.1	F: CCTTGTACTCGACAGGGACG R: CTGCAGTTTCAGCTCTCGGA	103
*β-actin*	NM_001310421.1	F: GATGGCTCCGGTATGTGCAA R: CAACCATCACACCCTGATGTC	103

FSHR, follicle stimulating hormone receptor; LHR, luteinizing hormone receptor; ESR2, estrogen receptor.

**Table 4:** Changes in fiber and active compound content Chinese herbal medicine

Item	Crushed CHM	fermented CHM	Enzymatically fermented CHM	SEM	p-value
NDF (%)	27.90^a^	27.04^a^	25.00^b^	0.467	0.003
ADF (%)	10.79^a^	9.82^b^	9.42^b^	0.240	0.022
Total flavonoids (mg/g)	0.06^a^	0.09^b^	0.09^b^	0.004	<0.001
Crude polysaccharide (%)	9.22	8.24	8.99	0.186	0.050
Total saponin (mg/g)	0.61^a^	0.70^b^	0.87^c^	0.039	<0.001

CHM, Chinese herbal medicine; SEM, standard error of the means; NDF, neutral detergent fiber; ADF, acid detergent fiber.

a–c Values within a row with different superscripts differ significantly at p<0.05.

**Table 5:** Effect of enzymolysis fermentation of Chinese herbal medicine on egg production performance in laying hens

Items	C	CT	FC	EFT	SEM	p-value
Laying rate (%)						
1 to 4 wk	78.22^a^	77.23^a^	81.34^b^	82.59^b^	0.640	<0.001
5 to 8 wk	76.17^a^	75.79^a^	80.27^b^	82.87^c^	0.800	<0.001
Egg weight (g)						
1 to 4 wk	62.93	62.78	63.41	63.96	0.176	0.056
5 to 8 wk	63.12	63.03	63.32	64.16	0.227	0.293
Feed intake (g)						
1 to 4 wk	122.20	123.40	124.87	125.36	1.013	0.725
5 to 8 wk	126.93^a^	129.94^b^	131.34^bc^	132.87^c^	0.663	<0.001
FCR						
1 to 4 wk	2.55^a^	2.55^a^	2.38^b^	2.36^b^	0.031	0.013
5 to 8 wk	2.73^a^	2.71^a^	2.44^b^	2.40^b^	0.039	<0.001

C, control group; CT, crushed Chinese herbal medicine group; FC, fermented Chinese herbal medicine group; EFT, enzymatically fermented Chinese herbal medicine group; SEM, standard error of the means; FCR, feed conversion ratio.

a–c Values within a row with different superscripts differ significantly at p<0.05.

**Table 6:** Effect of enzymolysis fermentation of Chinese herbal medicine on egg quality in laying hens

Items	C	CT	FC	EFT	SEM	p-value
Haugh unit						
1 to 4 wk	74.45	74.49	74.15	74.51	0.172	0.893
5 to 8 weeks	73.35^a^	74.91^ab^	75.36^bc^	76.88^c^	0.416	0.009
Albumin height (mm)						
1 to 4 wk	6.32	6.34	6.34	6.35	0.007	0.425
5 to 8 wk	6.24^a^	6.29^a^	6.37^b^	6.42^c^	0.019	<0.001
Yolk color						
1 to 4 wk	5.68	5.67	5.67	5.74	0.012	0.135
5 to 8 wk	5.52^a^	5.68^b^	5.70^b^	5.78^b^	0.030	0.006
Shell thickness (mm)						
1 to 4 wk	0.373^a^	0.376^ab^	0.379^ab^	0.382^b^	0.001	0.034
5 to 8 wk	0.357^a^	0.364^ab^	0.371^b^	0.383^c^	0.002	<0.001
Shell strength (N)						
1 to 4 wk	39.49	39.16	38.99	38.84	0.148	0.493
5 to 8 wk	37.44^a^	38.98^ab^	39.36^b^	40.49^b^	0.369	0.012
Egg shape index						
1 to 4 wk	1.31	1.32	1.30	1.31	0.002	0.150
5 to 8 wk	1.31	1.31	1.31	1.31	0.002	0.243

C, control group; CT, crushed Chinese herbal medicine group; FC, fermented Chinese herbal medicine group; EFT, enzymatically fermented Chinese herbal medicine group; SEM, standard error of the means.

a–c Values within a row with different superscripts differ significantly at p<0.05.

**Table 7:** Effect of enzymolysis fermentation of Chinese herbal medicine on serum lipid metabolism in laying hens

Item	C	CT	FC	EFT	SEM	p-value
TC (mmol/L)	2.63^a^	2.62^a^	2.12^b^	1.82^c^	0.106	<0.001
TG (mmol/L)	9.70	9.76	9.70	9.73	0.128	0.999
ALP (U/L)	241.33	247.00	235.00	233.33	2.156	0.066

C, control group; CT, crushed Chinese herbal medicine group; FC, fermented Chinese herbal medicine group; EFT, enzymatically fermented Chinese herbal medicine group; SEM, standard error of the means; TC, total cholesterol; TG, triglyceride; ALP, alkaline phosphatase.

a–c Values within a row with different superscripts differ significantly at p<0.05.

**Table 8:** Effect of enzymolysis fermentation of Chinese herbal medicine on serum hormones in laying hens

Item	C	CT	FC	EFT	SEM	p-value
E2 (pg/mL)	335.45	338.08	335.08	335.18	2.807	0.980
LH (mIU/mL)	9.13^a^	9.27^a^	9.70^a^	10.67^b^	0.153	<0.001
FSH (mIU/mL)	12.54	12.55	12.51	12.63	0.119	0.990

C, control group; CT, crushed Chinese herbal medicine group; FC, fermented Chinese herbal medicine group; EFT, enzymatically fermented Chinese herbal medicine group; SEM, standard error of the means; E2, estrogen; LH, luteinizing hormone; FSH, follicle stimulating hormone.

a,b Values within a row with different superscripts differ significantly at p<0.05.

**Table 9:** Effect of enzymolysis fermentation of Chinese herbal medicine on the mRNA expression of ovarian hormone receptors in laying hens

Item	C	CT	FC	EFT	SEM	p-value
FSHR	1.00^a^	1.39^b^	1.60^c^	1.98^d^	0.054	<0.001
LHR	1.00^a^	1.46^b^	1.97^c^	2.68^d^	0.092	<0.001
ESR2	1.00^a^	0.99^a^	1.43^b^	1.49^b^	0.036	<0.001

C, control group; CT, crushed Chinese herbal medicine group; FC, fermented Chinese herbal medicine group; EFT, enzymatically fermented Chinese herbal medicine group; SEM, standard error of the means; FSHR, follicle stimulating hormone receptor; LHR, luteinizing hormone receptor; ESR2, estrogen receptor 2.

a–d Values within a row with different superscripts differ significantly at p<0.05.
